# Time-sensitive changes in the maternal brain and their influence on mother-child attachment

**DOI:** 10.1038/s41398-024-02805-2

**Published:** 2024-02-09

**Authors:** Susanne Nehls, Elena Losse, Christian Enzensberger, Thomas Frodl, Natalia Chechko

**Affiliations:** 1 Department of Psychiatry, Psychotherapy and Psychosomatics, Medical Faculty, RWTH Aachen, Aachen, Germany; 2 Institute of Neuroscience and Medicine: JARA-Institute Brain Structure Function Relationship (INM-10), Research Center Jülich, Jülich, Germany; 3 Department of Gynecology and Obstetrics, Medical Faculty, RWTH Aachen, Aachen, Germany; 4 Department of Psychiatry and Psychotherapy, Faculty of Medicine, Otto von Guericke University Magdeburg, Magdeburg, Germany; 5 German Center for Mental Health (DZPG), Center for Intervention and Research on Adaptive and Maladaptive Brain Circuits Underlying Mental Health (C-I-R-C), Jena-Magdeburg-Halle, Germany; 6 Institute of Neuroscience and Medicine, Brain and Behavior (INM-7), Research Center Jülich, Jülich, Germany

**Keywords:** Neuroscience, Diseases

## Abstract

Pregnancy and the postpartum period are characterized by an increased neuroplasticity in the maternal brain. To explore the dynamics of postpartum changes in gray matter volume (GMV), magnetic resonance imaging was performed on 20 healthy postpartum women immediately after childbirth and at 3-week intervals for 12 postpartum weeks. The control group comprised 20 age-matched nulliparous women. The first 6 postpartum weeks (constituting the subacute postpartum period) are associated with decreasing progesterone levels and a massive restructuring in GMV, affecting the amygdala/hippocampus, the prefrontal/subgenual cortex, and the insula, which approach their sizes in nulliparous women only around weeks 3–6 postpartum. Based on the amygdala volume shortly after delivery, the maternal brain can be reliably distinguished from the nulliparous brain. Even 12 weeks after childbirth, the GMV in the dorsomedial prefrontal cortex, and the cortical thickness of the subgenual and lateral prefrontal cortices do not reach the pre-pregnancy levels. During this period, a volume decrease is seen in the cerebellum, the thalamus, and the dorsal striatum. A less hostile behavior toward the child at 6–12 weeks postpartum is predicted by the GMV change in the amygdala, the temporal pole, the olfactory gyrus, the anterior cingulate, the thalamus and the cerebellum in the same period. In summary, the restructuring of the maternal brain follows time-dependent trajectories. The fact that the volume changes persist at 12 weeks postpartum indicates that the maternal brain does not fully revert to pre-pregnancy physiology. Postpartum neuroplasticity suggests that these changes may be particularly significant in the regions important for parenting.

## Introduction

Pregnancy and the postpartum period are characterized by processes involving extensive physiological adaptation [1]. The increased hormone levels during pregnancy cross the blood-brain barrier and trigger changes in the brain architecture [2], affecting in particular the hippocampus, the amygdala, the subgenual cortex, the bilateral temporal lobe, the insula, the basal ganglia, and the cerebellum [3–5]. Following childbirth, the maternal postnatal adaptation processes involving structural change appear to begin quite early in the postpartum phase [6, 7], lasting up to 6 months [6] or even longer [4, 8]. The patterns of these changes have not yet been systematically investigated. Moreover, the disparate time points of investigation chosen by the studies in this field are likely to have influenced the results.

Pregnancy and the early postpartum period are critical for the establishment of a repertoire of maternal abilities suitable for the highly demanding task of caring for the newborn. Thus, structural changes during this period facilitate key aspects of motherhood in terms of adaptive restructuring [4, 9]. For instance, a GMV increase between the first and fourth months after childbirth has been found to relate to the absence of hostility (assessed by the Maternal Postnatal Attachment Scale [MPAS] [10]) toward the infant [4]. In contrast, in our previous study, we found no significant association between GMV at childbirth and MPAS total scores and sub-scores (attachment quality, pleasure in interaction, absence of hostility) at 3 or 12 weeks postpartum [3], which is in line with the recent study by Hoekzema et al. [5]. This discrepancy may suggest that the maternal brain is altered by pregnancy in such a way that its link to felt attachment develops during the process of motherhood and is likely to be influenced significantly by the interaction with the infant, e.g. [11]. To systematically understand the trajectories of post-childbirth brain structure restoration and their association with ovarian hormone levels, and to explore whether mother-infant attachment is linked to maternal neuroplasticity, we conducted a 12-week assessment, beginning in the first postpartum week and continuing at 3-week intervals. The assessments of mother-infant attachment and blood plasma estradiol and progesterone levels were performed concurrently with MRI measurements. Nulliparous women served as a control group.

We hypothesized that postpartum women would have lower GMV compared to their nulliparous counterparts at each time point of the study (hypothesis I). During the follow-up, we expected the mothers to have a continuous and widespread increase in GMV [2] (hypothesis IIb). We expected these changes to be not random, but strictly related to the investigation’s time frame (hypothesis IIb). Given the crucial role of the first 6 postpartum weeks in postpartum adaptation [12], we expected the most salient changes to occur within this period and affect particularly the areas linked to socio-emotional processing and emotion regulation (e.g., amygdala, hippocampus, prefrontal cortex) (hypothesis III). While a healthy brain may be an important prerequisite for appropriate attachment behavior, we expected no significant structural differences within the euthymic and mentally healthy sample [13]. Thus, based on our previous finding and insights from animal studies where pup interaction has been seen to be associated with neuroplasticity [11], we did not expect the GMV immediately after delivery to predict the development of attachment to the child at follow-up, assuming, instead, that the change in GMV over the 12-week follow-up would exhibit a correlation with attachment (hypothesis IV).

## Methods

Twenty-one non-depressed postpartum women were recruited within 1–7 days of childbirth in the Department of Gynecology and Obstetrics at University Hospital Aachen. Prior to enrollment in the study, written informed consent was obtained from each participant. The study, approved by the Ethics Committee of the University Hospital Aachen, conformed to the ethical standards of the Helsinki declaration. For further details of the recruitment procedure, please see Supplementary Materials and Methods.

Structural MRI measurements took place within 1 week postpartum, and then at 3, 6, 9 and 12 weeks postpartum. The average measurement time frames were 5.9 (SD = 2.17) days postpartum for T0, 22.45 days (SD = 2.39) for T1, 43.35 days (SD = 3.07) for T2, 64.55 (SD = 2.67) for T3 and 84.95 (SD = 3.49) for T4. At each time point, blood samples were drawn to determine the blood plasma estradiol and progesterone levels. The participants completed the Edinburgh Postnatal Depression Scale (EPDS) [14] immediately after childbirth and every 3 weeks for 12 weeks postpartum. Within 1 week postpartum, the participants indicated the symptoms of baby blues during the first postpartum week by means of the Maternal Blues Questionnaire (MBQ) [15]. Using the MPAS [10], a total score of maternal attachment including sub-scores for quality of attachment, absence of hostility and pleasure in interaction was evaluated at 3, 6, 9 and 12 weeks postpartum.

The data of one participant were excluded due to high values indicating severe depressive symptoms throughout the postpartum period. The data of 20 postpartum women were used for analyses (mean age = 32.25, SD = 3.97, age range = 24–39).

### Nulliparous control subjects

The imaging data of nulliparous control subjects with no prior pregnancy, regardless of pregnancy outcome (e.g., extrauterine pregnancy, or spontaneous, medical or surgical abortion) and with no history of psychiatric disorders were selected from a data pool of the study center (*n* = 67). We used case-control matching with SPSS 27 to perform age (years) matching between nulliparous controls and postpartum women at the time of delivery. The 1:1 matching approach with a tolerance of 0 as a starting point, randomizing the order of cases when drawing the matched pairs, resulted in 18 matched pairs. For cases that did not match exactly, we allowed for a tolerance of ±1 year, ensuring that both groups exhibited similar age distributions, means, and standard deviations. The data of 20 control subjects were thus used for analyses (mean age = 32.05, SD = 3.20, age range = 24–39). There were no significant age differences between the nulliparous and postpartum groups (*t*(38) = 0.174, *p* = 0.863).

### Hormonal assays

Progesterone and estradiol serum concentrations were measured before each scanning session and analyzed by competitive immunometry electrochemistry luminescence detection at the Laboratory Diagnostic Center, University Hospital Aachen. The samples were run on a Roche Cobas e601 and on a Roche Cobas e801 with Cobas Elecsys estradiol and progesterone reagent kits, respectively (Roche Diagnostics, Bromma, Sweden). For progesterone, the measurement interval was 0.05–60 ng/ml with an intra-assay coefficient of variation (CV) of 2.33–2.49%. For estradiol, the measurement interval was 5–3000 pg/ml with an CV of 1.77–2.91%.

Progesterone and estradiol levels were missing for one participant at 9 weeks postpartum.

### Behavioral data analyses

The analysis was conducted using SPSS® 27 IBM Corporation, Armonk NY, USA for Windows®.

Shapiro–Wilk tests revealed a positively skewed distribution of progesterone and estradiol levels. To reach normally distributed values, natural logarithmic transformation was performed.

Analyses of variance (ANOVA) for repeated measures were calculated with measurement time points after delivery and EPDS scores, MPAS total scores and subscale scores, or log-transformed hormone levels as within-subject variables. The Greenhouse-Geisser correction was used to adjust degrees of freedom when significant non-sphericity was detected via the Mauchly’s test. The effect sizes of the significant results are reported using partial eta squared (ηp2) for *F*-tests (small: 0.02–0.05, medium: 0.06–0.013, large: 0.14 and greater) (Cohen 1988) [16].

Exact Fisher test was performed to test the association of breastfeeding behavior and resumed menstruation at 12 weeks postpartum. Association of breastfeeding behavior and log-transformed hormone levels were carried out using Spearman’s rank correlation.

A significance level of *p* value < 0.05 was used.

### MRI data acquisition and analysis

Neuroimaging data were acquired using a 3 Tesla Prisma MR Scanner (Siemens Medical Systems, Erlangen, Germany) located in the Medical Faculty of RWTH Aachen University. T1-weighted structural images were acquired by means of a 3-dimensional magnetization-prepared rapid acquisition gradient echo imaging sequence.

Imaging data were preprocessed using the Computational Anatomy Toolbox (CAT12 Version r1872) and statistical parametric mapping (SPM)12 toolbox implemented in Matlab 2015b (MathWorks, Inc., Natick, MA). For the cross-sectional comparison of nulliparous women and postpartum women, default settings of cross-sectional analysis of CAT12 were applied for spatial registration, segmentation, and normalization [17]. For longitudinal analyses of the postpartum women, the structural images were processed according to the longitudinal protocol in CAT12. Additional information on cortical thickness and sulcus depth was extracted.

For details of the recording protocol and preprocessing of the structural data, please see Supplementary Materials and Methods.

### Analyses of VBM and SBM

For the cross-sectional comparison, we applied independent *t*-tests for the nulliparous group vs. each postpartum time point. For voxel-based morphometry (VBM) analyses, age and total intracranial volume (TIV) were used as covariates, while for surface-based morphometry (SBM) analyses only age was used as a covariate [17].

For longitudinal analyses of the postpartum GMV change, we used a flexible-factorial general linear model with the factors subject and time points. Significant effects were further pursued with t-contrast. For the VBM analysis, total intracranial volume (TIV) was used as a covariate. The CAT12 manual [17] does not recommend the use of covariates that do not change over time, which is age.

Unless otherwise mentioned, for all analyses, the statistical threshold was set at *p* < 0.05 cluster-level family-wise error (FWE) correction for multiple comparisons, with a cluster-forming threshold at voxel-level *p* < 0.001. Gray matter structures in the VBM analyses are labeled with reference to the Automated anatomical labeling atlas 3 [18]. In SBM analyses, surface structures are labeled with reference to the Desikan–Killiany atlas [19]. All results are presented in the MNI space.

### Multivariate regression analyses

We performed a multivariate pattern recognition analysis using the Pattern Recognition for Neuroimaging Toolbox 3.0 (PRoNTo [20]), a user-friendly tool providing recommendations tailored to the specific research question and dataset, in order to examine whether postpartum women could be distinguished from their nulliparous counterparts on the basis of the whole-brain GMV. Following the recommendation in the user’s manual, and in order to be able to replicate the analyses of Hoekzema et al. [4, 5] we applied an L1 multiple-kernel learning (MKL) algorithm, which considers each of the modality-specific kernels to build the classifier model. A nested cross-validation (CV) with hyperparameter optimization was used for assessment of the generalization error (soft-margin C ranged 0.01, 0.1, 1, 10, 100, 1000). For the inner loop, 5-fold CV on subjects-per-group and for the outer loop, 10-fold CV on subjects-per-group were used. Features were mean-centered and normalized, and age was regressed out. Model performance was assessed by balanced accuracy (BAC) values, specificity, sensitivity, and predictive values for each classifier. Statistical significance of classification accuracy was determined by permutation tests repeated 1000 times. Additionally, the spatial representation of the predictive function (i.e., weight maps), was estimated and were labeled according to the Anatomical Automatic Labeling (AAL) atlas. Regions were ranked according to their contribution to the model and averaged across folds.

To examine whether GMV change across the postpartum period predicts maternal attachment, we first calculated GMV change images by subtracting consecutive time points (e.g., 12 weeks minus 9 weeks) of each individual with the ImCalc function in SPM 12. The PRoNTo toolbox 3.0 [20] was then used to predict the MPAS total score and the subscales Quality of Attachment, Absence of Hostility, and Pleasure in Interaction using the whole-brain voxel-wise GMV change images, controlling for age. Based on the previous analyses of Hoekzema et al. [4, 5] and following the recommendation in the user’s manual, a kernel ridge regression was computed using a leave-one-out approach with the default settings. Model accuracy was quantified with the correlation (Pearson’s *r*), total variance explained (*R*^2^), and root mean squared error (RMSE). Permutation-based non-parametric *p* values (1000 permutations) were computed for the correlation between observed and predicted scores.

## Results

Socio-economic characteristics and information regarding obstetric and postpartum variables can be found in Supplementary Table S1. Table 1 summarizes the mean values of baby blues, depressivity and maternal attachment scores throughout the postpartum period.

**Table 1:** Means and standard deviation of maternal attachment, depressivity and baby blues scores in the postpartum period.

	Mean (SD)
MPAS 3 weeks pp	80.9 (10.15)
Quality of attachment	40.85 (4.51)
Absence of hostility	18.5 (3.33)
Pleasure in interaction	21.55 (3.79)
MPAS 6 weeks pp	83.85 (7.88)
Quality of attachment	42.3 (3.50)
Absence of hostility	19.7 (2.70)
Pleasure in interaction	21.85 (3.31)
MPAS 9 weeks pp	83.45 (8.82)
Quality of attachment	42.5 (3.04)
Absence of hostility	19.4 (3.60)
Pleasure in interaction	21.55 (3.91)
MPAS 12 weeks pp	83.95 (8.32)
Quality of attachment	43.1 (2.15)
Absence of hostility	19 (3.78)
Pleasure in interaction	21.85 (4.32)
EPDS within 1 week pp	6.8 (4.35)
EPDS 3 weeks pp	6.9 (5.31)
EPDS 6 weeks pp	5.3 (3.48)
EPDS 9 weeks pp	4.45 (3.15)
EPDS 12 weeks pp	3.45 (2.86)
MBQ within 1 week pp	11.30 (6.56)

*MPAS* Maternal Postnatal Attachment Scale, *EPDS* Edinburgh Postnatal Depression Scale, *MBQ* Maternity Blues Questionnaire, *pp* postpartum.

Throughout the postpartum period, the participants had significantly decreasing EPDS scores (*F*(2.43, 46.09) = 73.56, *p* = 0.003, eta^2^ = 0.240), while maternal attachment toward the child increased over time (*F*(3, 57) = 3.138, *p* = 0.032, eta^2^ = 0.142). The latter was particularly driven by the increased felt attachment (*F*(1.92, 36.48) = 4.038, *p* = 0.027, eta^2^ = 0.175), while absence of hostility and pleasure in interaction remained stable.

At 12 weeks postpartum, 14 women were actively breastfeeding their infant and 7 women indicated the resumption of menstruation while 10 had no menstruation yet (3 missing answers). Breastfeeding at 12 weeks and resumed menstruation at 12 weeks were not significantly associated (Exact Fisher *p* = 0.060). Breastfeeding was negatively correlated with log-transformed estradiol and progesterone levels at 9 weeks (estradiol *r* = −0.644, *p* = 0.003; progesterone: *r* = −0.474, *p* = 0.035) and 12 weeks (estradiol *r* = −0.483, *p* = 0.036; progesterone: *r* = −0.617, *p* = 0.004). No correlation was observed between the MBQ scores and the progesterone and estradiol levels within the first week of childbirth.

**Fig. 1:**
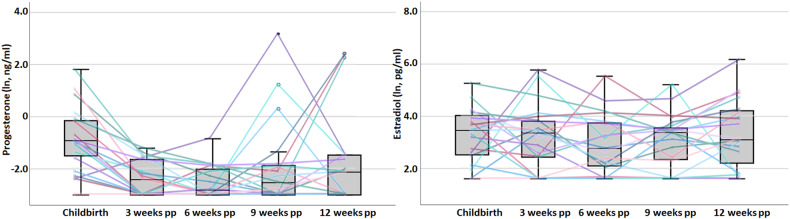
Boxplots of natural log-transformed (ln) mean plasma concentration of estradiol and progesterone with 25th and 75th percentile as well as minimum and maximum. The lines represent the plasma concentration of the hormones on the respective sampling day for each individual.

Only for progesterone, a significant interaction with time was observed (*F*(2.38, 42.77) = 5.23, *p* = 0.006, eta^2^ = 0.225) following a quadratic curve (*p* = 0.002) (see Fig. 1). After removing 3 women with outliers at 9 weeks and 3 women with outliers at 12 weeks, the significant interaction with time (*F*(2.12, 25.39) = 21.95, *p* < 0.001, eta^2^ = 0.646) followed both a linear trend (*p* = 0.001) with significantly decreasing values, and a quadratic curve (*p* < 0.001) with leveling values at 6 and 9 weeks and a slight increase at 12 weeks (Table S2 and Fig. S1).

### MRI results

#### Results of whole-brain voxel-based analyses (VBM)

##### Comparison between the postpartum and nulliparous brains

**Fig. 2:**
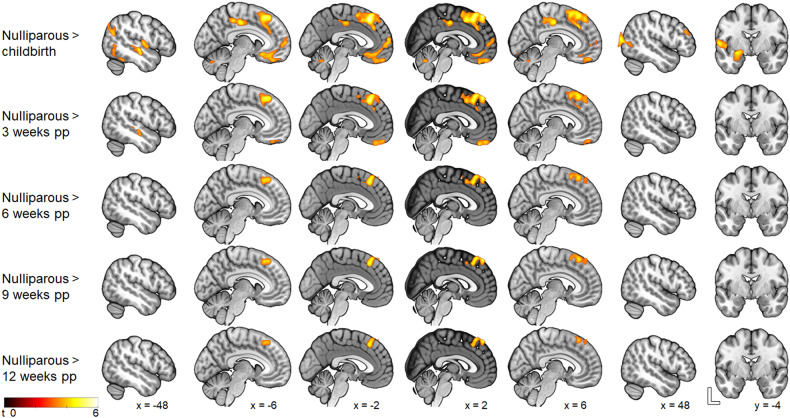
Smaller gray matter volume in postpartum women compared to nulliparous participants throughout the postpartum period.

Using t-contrast, we found a pattern of significantly smaller GMV in the left amygdala, the hippocampus, the parahippocampal gyrus, the pallidum and the putamen in the postpartum group at childbirth compared to the nulliparous group. Further smaller GMV was visible in the left superior frontal gyrus, the rectal gyrus, the middle orbital gyrus (Brodmann area 25), the bilateral posterior medial frontal gyrus, the anterior and midcingulate cortices, as well as the precuneus, the bilateral middle and superior temporal gyri, the bilateral inferior occipital gyrus and the cerebellum (crus1, crus 2, VI, VII) (Fig. 2 and Table S3).

At 3 weeks postpartum, compared to the nulliparous group, smaller GMV was found in the bilateral posterior and superior medial frontal gyri, the bilateral MCC, the left middle and superior temporal gyri, the right rectal gyrus and the superior orbital gyrus of the mothers. At both 6, 9, and 12 weeks postpartum, the postpartum brain showed consistently smaller GMV in the bilateral superior and posterior medial frontal gyri (Fig. 2 and Table S3).

Compared to the nulliparous participants, the postpartum group did not show significantly greater GMV.

##### GMV alteration in the postpartum period

**Fig. 3:**
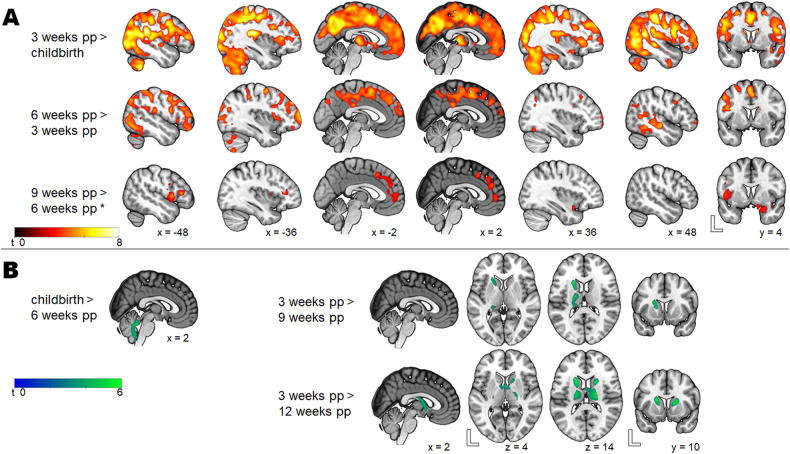
Postpartum longitudinal changes in gray matter volume. **A** Postpartum gray matter volume increase at 3 weeks postpartum compared to childbirth, 6 weeks compared to 3 weeks and 9 weeks compared to 6 weeks. *The comparison 9 weeks vs. 6 weeks postpartum was performed with cluster-forming threshold to *p* < 0.005, using a *p* < 0.05 cluster-level FWE-correction. **B** Postpartum gray matter volume decrease from childbirth to 6 weeks postpartum and from 3 weeks to 9 and 12 weeks postpartum.

The greatest GMV increase occurs within the first 3 weeks postpartum in the bilateral prefrontal, parietal, temporal, and occipital cortices and the insula (Fig. 3 and Table S4). The next 3 weeks (6 weeks compared to 3 weeks postpartum) were characterized by less volume enlargement but were likewise widespread in all lobes of the cerebral cortex (Fig. 3 and Table S4). At 9 weeks compared to 6 weeks postpartum, only the right inferior frontal gyrus and the insula gained more volume. However, an exploratory reduction of the cluster-forming threshold to *p* < 0.005, using a *p* < 0.05 cluster-level FWE-correction, revealed even further increases in the left and right superior medial gyri, the left middle orbital gyrus and the left anterior cingulate gyrus, as well as in the right amygdala and the right hippocampus.

No significant volume increase was detected at 12 weeks compared to 9 weeks postpartum.

To account for prior reports in the literature showing GMV increase at 12 weeks postpartum, we sought to determine whether there was GMV increase from 6 to 12 weeks postpartum and found a significant increase in the medial prefrontal cortex, including the anterior and midcingulate cortices, the bilateral temporal cortex as well as the left fusiform gyrus, the amygdala, the hippocampus and the parahippocampal gyrus.

To test whether there was a GMV decrease in the postpartum phase, we contrasted the neighboring time points (e.g., childbirth vs. 3 weeks, 3 vs. 6 weeks, 6 vs. 9 weeks, 9 vs. 12 weeks) and found no significant GMV decrease. A comparison of the distal time points, however, showed GMV decrease in the bilateral cerebellum lobule IX from childbirth to 6 weeks postpartum, as well as in the bilateral caudate, the putamen and the thalamus from 3 to 12 weeks.

##### Classification between nulliparous and postpartum women

**Fig. 4:**
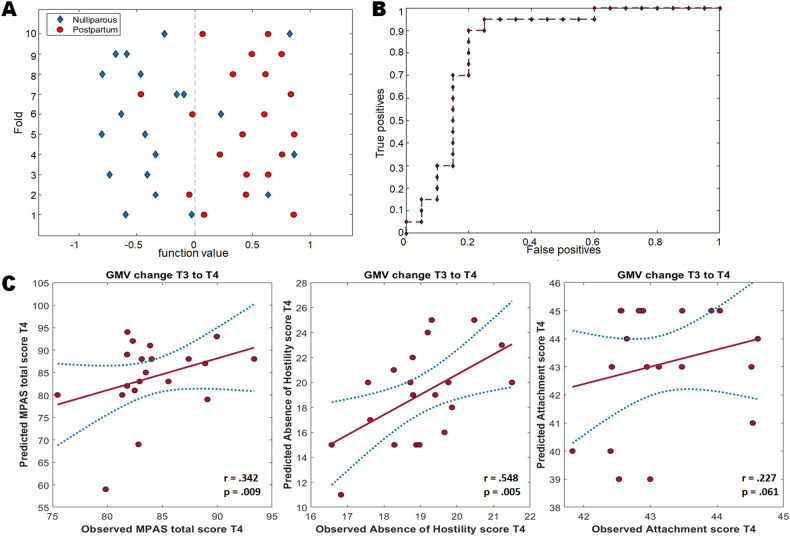
Multivariate regression analyses. **A** Classification plot comparing nulliparous and postpartum women. Positive function values for postpartum women indicates true positives. Negative function values for nulliparous participants indicates true negatives. **B** Receiver operating characteristic (ROC) curve showing classification performance. **C** Gray matter volume change from 9 weeks to 12 weeks postpartum predicts the total score of the Maternal Postnatal Attachment scale, the score of the subscale Absence of Hostility, and, by trend, the score of the subscale Quality of Attachment.

The MKL analysis enabled the classification between nulliparous and postpartum women at childbirth with a significant total BAC of 82.5% (*p* = 0.004, permutation *n* = 1000), postpartum sensitivity of 85% (permutation *n* = 1000, *p* = 0.002), nulliparous specificity of 80% (permutation *n* = 1000, *p* = 0.019) and the AUC of 0.78 (*p* < 0.001) (see Fig. 4A, B, density plot of permutation test in Fig. S2A). The class predictive value was 83.33% for the postpartum group and 86.67% for the nulliparous group. The region with the greatest predictive power was the left amygdala (weight 86.6%, experimental ranking [ER] = 113), followed by the left Heschl gyrus (weight 2.96%, ER = 22.7), the right pallidum (weight 2.03%, ER = 22.5), right cerebellum IX (weight: 1.65%, ER = 22), the right rectal gyrus (weight: 1.51%, ER = 11.5) and the left cerebellum VI (weight 1.18, ER = 11.5).

The classification between nulliparous and postpartum participants at 3 (BAC = 52.5%, *p* = 0.356), 6 (BAC = 42.5%, *p* = 0.657), 9 (BAC = 40%, *p* = 0.774) and 12 weeks postpartum (52.5%, *p* = 0.264) did not prove statistically significant.

##### Association between postpartum GMV and maternal attachment

Using multiple regression analyses, we first tested whether the whole-brain GMV correlated with the concurrent MPAS total scores and sub-scores at each time point. No significant correlation was found using a *p* < 0.05 cluster-level FWE-correction, with a cluster-forming threshold at voxel-level *p* < 0.001. and nor at a less conservative voxel level *p* < 0.005.

We used Kernel ridge regression to test whether the MPAS total score and its sub-scales (quality of attachment, absence of hostility, and pleasure in interaction) at any time point are predicted by the GMV change from 3 weeks before to that time point, with TIV and age as covariates. Only the GMV increase from 9 to 12 weeks was found to predict the MPAS total score (*r* = 0.342, *p* = 0.009), the absence of hostility subscale (*r* = 0.548, *p* = 0.005), and by trend, the quality of attachment subscale (*r* = 0.227, *p* = 0.061). Pleasure in interaction was not predicted by the GMV change (*r* = −0.157, *p* = 0.166) (see Fig. 4C, density plot of permutation test in Fig. S2B).

The regions with the greatest contribution to the total MPAS score prediction were the cerebellar structures (Vermis 8, 7b, Crus1, 9, Crus2, 6, Crus1), followed by the left putamen and the left precuneus. For the subscale absence of hostility, the right amygdala, the left amygdala, the left middle temporal pole and the right superior temporal pole, the cerebellum (crus 1, crus 2, 9) and the left thalamus had the most predictive power. A full list of predictive regions with >1% contribution to the decision function for both the MPAS total score and the subscale absence of hostility can be found in the Supplementary Material (Table S5).

We also sought to determine whether the total score and the subscale scores were predicted by a cumulative GMV change from an earlier time point to T4, finding only the relevant change from 6 to 12 weeks postpartum to predict the absence of hostility (*r* = 0.34, *p* = 0.029). The greatest predictive power was found to stem from the left thalamus, the right superior temporal pole, the right olfactory gyrus, the cerebellum vermis 8, the Heschl gyrus, the left amygdala, the left anterior cingulate cortex and the right thalamus.

##### Association between postpartum GMV and mood symptoms

Using multiple regression analyses, we first tested whether the whole-brain GMV correlated with the concurrent MBQ and EPDS scores at each time point and found no significant correlation using a *p* < 0.05 cluster-level FWE-correction, with a cluster-forming threshold at voxel-level *p* < 0.001, and nor at a less conservative voxel level *p* < 0.005. Using Kernel ridge regression, we additionally tested whether (1) the GMV change from one time point to the other was associated with MBQ and EPDS at the later time point, and (2) whether GMV change and EPDS from one time point to the other correlate. No significant association was found for any scores at any time point.

##### Association between postpartum GMV and hormonal fluctuation

Only at 12 weeks postpartum, log-transformed progesterone levels correlated positively with GMV in the left inferior frontal gyrus pars orbitalis and the left middle orbital gyrus. Estradiol levels did not correlate with GMV at any time point. Using Kernel ridge regression, we also tested whether (1) the GMV change from one time point to the other was associated with hormone levels at the later time point, and (2) whether GMV change and hormone level change from one time point to the other correlate. No significant association was found for any hormone at any time point.

#### Results of whole-brain surface-based analyses (SBM)

##### Differences in cortical thickness between nulliparous and postpartum brains

**Fig. 5:**
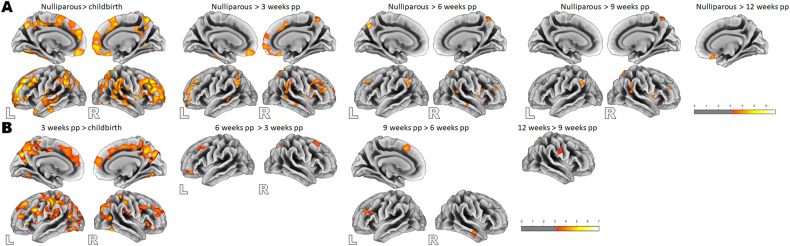
Changes in cortical thickness. **A** Greater cortical thickness in nulliparous women compared to postpartum women at all study time points (pp). **B** Increase of cortical thickness in the postpartum period.

We assessed the structural differences between the maternal and nulliparous brains by means of surface-based analyses. In line with the volumetric analyses, we found reduced cortical thickness across the postpartum period compared to the nulliparous group, with the greatest difference being at childbirth and then diminishing at the subsequent measurement time points (see Fig. 5A and Table S6). The opposite contrasts revealed no greater cortical thickness in the postpartum group compared to the nulliparous participants.

##### Cortical thickness alteration in the postpartum period

Examining the surface characteristics across the postpartum period itself, we found an increase in cortical thickness from childbirth to 3 weeks postpartum in both hemispheres in the lateral and medial prefrontal cortices including the cingulate cortex, the parietal cortex, the posterior part of the temporal cortex and fusiform gyri, and the insular cortex (Fig. 5B and Table S7). From 3 to 6 weeks postpartum, an increase in cortical thickness was found in the left middle frontal and inferior frontal gyri, and the right superior frontal gyrus and the superior parietal lobule (Fig. 5B and Table S7). No increase was found from 6 to 9 weeks and 9 to 12 weeks postpartum. However, using a *p* < 0.05 cluster-level FWE-correction, with a cluster-forming threshold at voxel-level *p* < 0.005, increased cortical thickness from 9 to 12 weeks was found in the right pre- and postcentral gyri extending to the supramarginal gyrus (Fig. 5B and Table S7). Furthermore, contrasting the more distal time points, 12 weeks vs. 6 weeks postpartum, cortical thickness increase was observed in the right superior frontal, middle frontal and inferior frontal gyri as well as in the left middle and inferior temporal gyri.

Comparing the time points of childbirth and 12 weeks postpartum, a decrease in cortical thickness throughout the postpartum period was found in the left medial orbital gyrus and the right lingual gyrus (see Supplementary Material).

##### Sulcus depth

Changes in the surface area (i.e., sulcus depth reduction) were seen at all time points during the postpartum follow-up, with greatest reduction being at 3 weeks compared to childbirth, and 6 weeks compared to 3 weeks, particularly in the lateral prefrontal and temporal cortices, the parietal cortex and the insula (see Supplementary Fig. S3 and Table S8). At 9 weeks compared to 6 weeks, the left temporal cortex, and 12 weeks compared to 9 weeks, the right inferior frontal sulcus displayed a decreasing depth (see Supplementary Fig. S3 and Table S8). No differences in sulcus depth were seen in nulliparous participants.

## Discussion

In a healthy postpartum sample recruited within 1 week of childbirth, we found, in line with our previous analyses (Chechko et al. [3]), that, shortly after delivery, maternal brains are profoundly different from those of nulliparous women and can be reliably classified based on GMV changes particularly in the left amygdala. Further postpartum morphological restructuring was seen to follow time-sensitive trajectories, with major changes occurring during the subacute postpartum period.

### The significance of the first 6 postpartum weeks: the subacute postpartum period

The subacute postpartum period is suggested to last 2–6 weeks and is characterized by major changes in terms of hemodynamics, genitourinary recovery, metabolism, and emotional status [12]. Accordingly, the most significant processes, both in terms of smaller GMV compared to nulliparous women and the GMV and cortical thickness increase during the follow-up in the postpartum group, were observed from childbirth until the end of the subacute postpartum period (i.e., 6 weeks). The neuroplasticity processes were found to affect the volume and cortical thickness of the subgenual prefrontal cortex, the cingulate cortex, the insula, as well as the volume of the bilateral amygdala and the hippocampus, which approached the size of nulliparous women only toward weeks 3 to 6 postpartum. These regions are thought to be linked to the socio-emotional processing and regulation of stress [21, 22]. It has been suggested, also, that in postpartum women, the activation of the subgenual anterior cingulate is influenced by the physiological stress during pregnancy [23], differing between women who experience baby blues and those who do not [24]. The observed GMV enlargement during the postpartum period was paralleled by very low levels of progesterone, which were found to keep decreasing following childbirth, remaining so for all participants up until week 6. The progesterone fluctuation is thought to be related to baby blues symptoms [1, 25], which usually disappear after 2 weeks at the latest and affect only those at risk for hormone-related affective disorders [26]. That no correlation was seen between baby blues and GMV in the first week after childbirth suggests that baby blues symptoms do not necessarily exert an additive influence on morphology, as volumetric differences are already induced by the underlying physiological processes.

### Neuroplasticity during the delayed postpartum period

The following weeks, i.e., 6–12 weeks postpartum, were characterized by slightly increasing levels of progesterone, suggesting the recovery of ovarian function [27]. Although not too many changes were found to occur in the GMV and the surface even in 12 weeks after childbirth, it appears that GMV in the dorsomedial prefrontal cortex, and the cortical thickness of the subgenual prefrontal cortex and the lateral prefrontal cortex do not reach pre-pregnancy levels, corroborating the results of studies that have found differences between 2 and 6 years after childbirth [4, 5, 8]. Interestingly, within this time frame (between 6 and 12 weeks), both an increase and a decrease of GMV and cortical thickness were observed. Based on the only study to date that has found a volume reduction between 1 and 2 years after childbirth [28], it has been suggested that GMV decreases during pregnancy, increasing in the postpartum period until reaching baseline, and then decreasing again [29]. However, according to our observations, the GMV alteration follows a much more dynamic pattern, showing already from week 3 that the cerebellum, the thalamus and the dorsal striatum decrease in volume, with the surface of the orbital and lingual gyri becoming thinner. While the interpretation of these results may not be easy, potentially requiring further investigations, establishing a link to maternal behavior is possible given that maternal attachment is associated with the striatal dopamine function and the recruitment of a cortico–striatal–amygdala brain network, which augments the capability for attachment [30]. Furthermore, given that GMV change between 9 and 12 weeks postpartum was found to predict the extent of total maternal attachment (MPAS total score) as well as the absence of hostility, it seems that the maternal brain undergoes further maturation and specialization of the neural network subserving maternal behavior during the delayed postpartum period. While no significant volume changes were seen in the postpartum group between 9 and 12 weeks postpartum, the observed changes in sulcus depth, cortical thickness and GMV differences from nulliparous women suggest subtle yet relevant dynamic changes, which likely contribute to the development of maternal behavior and bonding.

#### Maternal brain: the role of the amygdala and hormonal dynamics

Although GMV differences within 1 week of childbirth were found to encompass all lobes of the brain, the classification performance between the maternal and nulliparous brains was overwhelmingly driven by the left amygdala, in addition to the left Heschl gyrus, the right pallidum, the right rectal gyrus and the cerebellum. The amygdala has been shown to express high densities of estradiol and progesterone receptors [31]. These hormones are thought to induce neuroplasticity primarily by modulating dendritic spine and synapse density [32]. Thus, the considerable shift of estradiol and progesterone during pregnancy [1] is likely involved in the volumetric change in the amygdala during pregnancy and the postpartum period, with the medial and the dorsal amygdala networks [30], including the striatum, the anterior and posterior cingulate, the ventromedial prefrontal cortex and the insula [33] being key elements in human maternal bonding. Less hostile behavior toward the child has been found to be predicted by the volumetric change of the bilateral amygdala, the temporal pole, the right olfactory gyrus, the left anterior cingulate, the bilateral thalamus and the cerebellum, regions involved in socio-emotional processing and social cognition [34–36]. The link to the olfactory function is intriguing given that the amygdala receives direct olfactory input [37], and olfaction plays an important role in the development of maternal behavior in humans [38–40]. The postpartum changes in brain structures involved in social processes may have an incremental adaptive benefit in refining abilities to recognize and respond to infant needs, identify social and threatening stimuli, or promote mother-infant attachment.

Various studies have found that ovarian sex hormones are relevant during brain development and play a role in sex differences in brain morphology [41]. In the present study, after removing outliers, we found the temporal trajectories of postpartum GMV changes to covary with the changes in progesterone levels, suggesting a correlation between endocrine and neurobiological changes. However, we found GMV to correlate only with progesterone in the left inferior frontal gyrus pars orbitalis and the left middle orbital gyrus. And the correlation was detected only at 12 weeks postpartum, thus at a time point when the ovarian function had already resumed [27]. While we do not conclude that no correlations exist between the postpartum brain structure and ovarian hormones, it is possible that postpartum neuroplasticity is not too closely linked to the postpartum hormone levels. Instead, neuroplasticity during pregnancy may be triggered by the exceedingly high hormone levels in pregnancy, resulting in a massive loss of volume in the cerebral structures. The very slow early-postpartum recovery of the hormone levels may therefore have no direct effect on early-postpartum neuroplasticity.

### Limitations and conclusion

One limitation worth mentioning is that the nulliparous control group was not measured as repeatedly as the postpartum group. However, we consider the comparisons to the nulliparous group to be robust given the unlikelihood of relevant changes in the nulliparous brain. Nevertheless, to increase statistical power, it would be interesting to also examine the nulliparous group longitudinally and at the same intervals multiple times and then compare the groups. Another limitation is the study’s observational character. It does not explain the neuronal mechanisms of volumetric changes due to altered glial proliferation or neurogenesis, changes in neuronal or glial size, cellular composition, and alterations of the extracellular space [42–46]. Indeed, in pregnancy, the biological, neuronal correlate of volumetric changes are not well understood and need further investigations. Finally, for the cross-sectional comparisons with the nulliparous group and correlational analyses, the group size of 20 postpartum women is relatively small. However, given that our main objective was to conduct a longitudinal study of postpartum neuroplasticity in a homogenous cohort of healthy postpartum women, the group size of 20 involving 6 repeated measurements is satisfactory. In addition, the application of machine learning to small groups such as our sample of postpartum women is prone to overfitting, likely leading to an overestimation of the model’s predictive abilities as the model is excessively tailored to the peculiarities of the small dataset [47–49]. The risk of overfitting and the specificity of the training data may thus contribute to an overly optimistic assessment of the generalizability of the model. In conclusion, while our study highlights the potential of machine learning in our specific context, the observed high accuracy rates and large correlations of the multivariate regression analyses should be interpreted with caution. That said, recent advances in the field of machine learning have introduced innovative techniques and methods to address the challenges associated with small sample sizes, showing new pathways for future research.

In conclusion, our study confirms that the postpartum morphological restructuring follows time-sensitive trajectories, with major changes occurring during the subacute postpartum period. These changes may be particularly significant in brain regions that play key roles in parenting behavior.

### Supplementary information

Supplemental Material

## Data Availability

The data of this study are not publicly available due to privacy and ethical restrictions. Data to support the findings of this study are available upon reasonable request.
