# Supplementary material for: Time-sensitive changes in the maternal brain and their influence on mother-child attachment
Source: Transl Psychiatry. 2024 Feb 9;14:84. doi: 10.1038/s41398-024-02805-2 (PMC10853535; doi:10.1038/s41398-024-02805-2)
Supplement: Supplementary file 1 — Supplemental Material [file 41398_2024_2805_MOESM1_ESM.docx]

**Supporting Information**

## **Recruitment procedure**

Beginning within one to seven days of childbirth, we recruited 21 postpartum mothers at University Hospital Aachen. Women with depression at the moment of recruitment, abuse of alcohol, drugs, psychotropic substances, antidepressant or antipsychotic medication during pregnancy, history of psychosis or manic episodes were excluded. Following informed consent, the participants were initially screened for signs of prenatal depression and were included in the study only when not diagnosed with clinical depression. Following informed consent, clinical-anamnestic screenings (demographic information, information about the pregnancy as well as individual and family psychiatric history) were carried out.

## **MRI Data Acquisition**

Neuroimaging data were acquired using a 3 Tesla Prisma MR Scanner (Siemens Medical Systems, Erlangen, Germany) located in the Medical Faculty of RWTH Aachen University. T1-weighted structural images were acquired by means of a 3-dimensional magnetization-prepared rapid acquisition gradient echo imaging sequence (4.12 min; 176 slices, TR = 2300 ms, TE = 1.99 ms, TI =900 ms, FoV= 256×256 mm2, flip angle = 9◦, voxel resolution = 1×1×1mm3). All images were inspected for structural abnormalities, scanner artifacts, and motion artifacts. In case of the latter two, imaging acquisition was repeated.

## **Voxel Based Morphometry (VBM)**

Imaging data were preprocessed using the Computational Anatomy Toolbox (CAT12 Version r1872) and statistical parametric mapping (SPM)12 toolbox implemented in Matlab 2015b (MathWorks, Inc., Natick, MA). All images were affine registered to standard tissue probability maps by correcting individual head positions and orientations and translated into Montreal Neurologic Institute (MNI) space. The acquired structural T1-weighted images at each time point were spatially normalized to Montreal Neurological Institute (MNI) space (resampled to a voxel size of 1.5×1.5×1.5mm), segmented into gray matter (GM), white matter (WM) and cerebrospinal fluid (CSF). Images were visually inspected for potential segmentation and registration errors. Following the suggestions of the CAT12 toolbox manual (Gaser et al. in review), a homogeneity check of the unsmoothed data identified no outliers, thus the GMVs of all participants were included in subsequent analyses. Finally, the modulated GMV was smoothed with an 8-mm full-width at half-maximum (FWHM) Gaussian kernel.

## **Surface-Based Morphometry (SBM)**

The CAT12 toolbox was used to extract information regarding cortical thickness. Volumes were segmented using surface and thickness estimation in the writing options. Local maxima were projected to the gray matter voxels by using neighbor relationship described by the WM distance, equaling cortical thickness. The estimation of cortical thickness was performed based on projection-based thickness including partial volume correction, sulcal blurring, and sulcal asymmetries without sulcus reconstruction (Dahnke et al. 2013). Topological correction was performed through an approach based on spherical harmonics. For interparticipant analysis, an algorithm for spherical mapping of the cortical surface was included (Yotter et al. 2011). An adapted volume-based diffeomorphic DARTEL algorithm was then applied to the surface for spherical registration. All scans were resampled and smoothed with a Gaussian kernel of 12-mm FWHM. The surface data were visually inspected for artifacts and all scans passed through the automatic surface data homogeneity check of the CAT12 toolbox

## **Results**

Table S1. Socio-demographic information of the postpartum women.

|  | *Mean (SD)* | *Percent* |
| --- | --- | --- |
| Age | 32.25 (3.97) |  |
| Gestational age in weeks | 38.98 (1.77) |  |
| Child’s birth weight (g) | 3182.75 (582.44) |  |
| Secondary education |  |  |
| Lowest (< 9 years) |  | 0 |
| Middle (10 -12 years) |  | 10 |
| Highest (> 13 years) |  | 85 |
| Birth mode |  |  |
| Spontaneous |  | 30 |
| Ventouse |  | 10 |
| C-section |  | 50 |
| Emergency C-section |  | 10 |
| Intend to breastfeed at T0 (yes) |  | 95 |
| Breastfeeding at T1 (yes) |  | 70 |
| Married (yes) |  | 75 |
| Single mother (yes) |  | 0 |
| Support at home by partner at T0 (school grades 1-6) | 1.8 (0.95) |  |
| Support at home by partner at T1 (school grades 1-6) | 2.0 (0.94) |  |

Table S2. Natural log-transformed (ln) mean plasma concentration of progesterone (ng/ml) and estradiol (pg/ml) with outliers (n = 19) and without outliers (n = 13)

|  | n = 19 | n = 13 |
| --- | --- | --- |
|  | *Mean (SD)* | *Mean (SD)* |
| Progesterone within one week pp | -0.84 (1.23) | -0.77 (1.13) |
| Progesterone 3 weeks pp | -2.36 (0.69 | -2.35 (0.71) |
| Progesterone 6 weeks pp | -2.52 (0.61) | -2.65 (0.46) |
| Progesterone 9 weeks pp | -1.89 (1.69) | -2.64 (0.47) |
| Progesterone 12 weeks pp | -1.65 (1.88) | -2.50 (0.59) |
| Estradiol within one week pp | 3.29 (1.05) | 3.42 (1.09) |
| Estradiol 3 weeks pp | 3.24 (1.25) | 2.91 (1.04) |
| Estradiol 6 weeks pp | 2.94 (1.12) | 2.58 (0.81) |
| Estradiol 9 weeks pp | 3.09 (1.03) | 2.63 (0.81) |
| Estradiol 12 weeks pp | 3.31 (1.33) | 2.87 (1.03) |


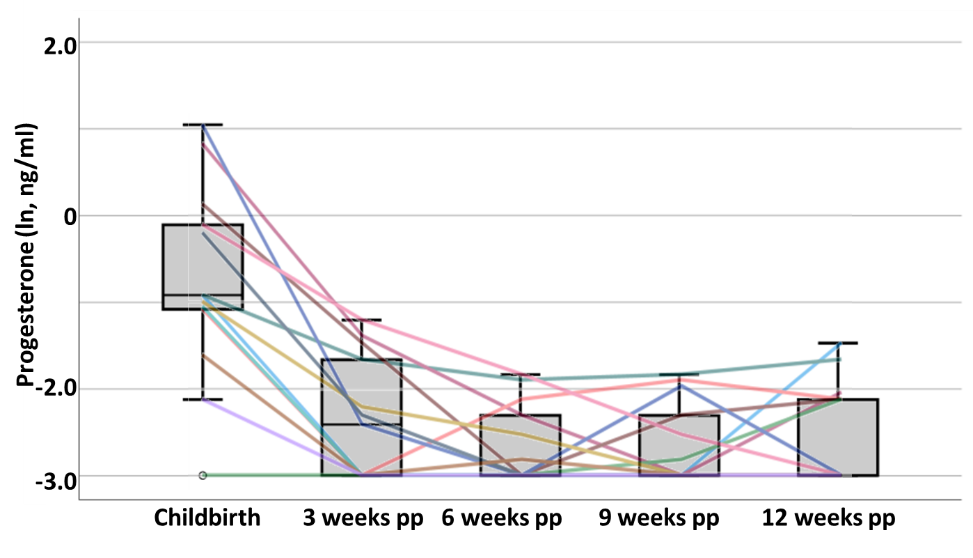


Figure S1. Boxplots of natural log-transformed (ln) mean plasma concentration of progesterone with 25^th^ and 75^th^ percentile as well as minimum and maximum after removal of outliers (n = 13 women). The lines represent the plasma concentration of the hormones on the respective sampling day for each individual.

Table S3. Brain regions showing gray matter volume in nulliparous women > postpartum women (t-contrast from random-effects GLM, p < .05, cluster-level FWE correction)

| **Anatomical Region** | **Brodmann** | **Side** | **size** | **T** | **x** | **y** | **z** |
| --- | --- | --- | --- | --- | --- | --- | --- |
| **Nulliparous > Postpartum childbirth** |  |  |  |  |  |  |  |
| Cerebellum 6, crus 1, fusiform gyrus, crus 2, inferior temporal gyrus, lingual gyrus, cerebellum 8, 7b, 4, middle temporal gyrus, inferior occipital gyrus, vermis 7, vermis 8, middle occipital gyrus, vermis 8 | 37, 18, 19, 20, 36 | L | 6113 | 5.05 | -40 | -56 | -21 |
| Middle temporal gyrus, rolandic operculum, angular gyrus, postcentral gyrus, Heschl gyrus, supramarginal gyrus, insula, middle occipital gyrus, inferior frontal gyrus p. opercularis | 22, 39, 21, 42, 6, 40, 43, 13, 41 | L | 4828 | 5.9 | -64 | -22 | -6 |
| Superior medial gyrus, supplementary motor area, midcingulate cortex, anterior cingulate cortex, superior frontal gyrus | 8, 6, 32, 9 | L/R | 4752 | 6.04 | -2 | 26 | 48 |
| Rectal gyrus, medial orbital gyrus, superior medial gyrus, olfactory cortex, superior orbital gyrus, anterior cingulate cortex | 11, 10, 25, 32, 47 | L/R | 2896 | 4.62 | -4 | 60 | 6 |
| Middle occipital gyrus, middle temporal gyrus, superior parietal lobule, precuneus, inferior occipital gyrus, angular gyrus | 19, 7, 39, 37, 18 | R | 2777 | 5.78 | 15 | -62 | 46 |
| Midcingulate cortex, paracentral lobule, supplementary motor area, precuneus | 24, 5, 7, 6 | L/R | 2708 | 6.35 | 10 | -34 | 40 |
| Cerebellum (VI, Crus 1, Crus 2, 8), lingual gyrus, fusiform gyrus | 18, 19 | R | 2455 | 4.67 | 22 | -74 | -24 |
| Superior temporal gyrus, middle temporal gyrus | 21, 22 | R | 895 | 4.58 | 64 | -24 | -6 |
| Middle frontal gyrus, inferior frontal gyrus p. triangularis | 10, 46, 9 | R | 873 | 5.08 | 38 | 39 | 21 |
| Amygdala, hippocampus, superior temporal pole, parahippocampal gyrus, putamen, pallidum, olfactory gyrus | 34, 28 | L | 839 | 4.82 | -26 | 2 | -20 |
| **Nulliparous > Postpartum 3 weeks** |  |  |  |  |  |  |  |
| Superior medial gyrus, supplemental motor area, midcingulate cortex, superior frontal gyrus, anterior cingulate cortex | 8, 6, 32, 9 | L/R | 3083 | 5.56 | -2 | 26 | 48 |
| Middle temporal gyrus, superior temporal gyrus | 21, 22 | L/R | 1055 | 5.26 | -64 | -22 | -6 |
| Cerebellum (Crus 1, 6, Crus 2, 8, 7b) |  | L | 876 | 4.07 | -16 | -68 | -38 |
| Rectal gyrus, superior orbital gyrus | 11 | R | 820 | 3.73 | 12 | 42 | -26 |
| **Nulliparous > postpartum 6 weeks** |  |  |  |  |  |  |  |
| Superior medial gyrus, supplementary motor area, superior frontal gyrus | 8, 6, 32 | L/R | 1834 | 5.15 | -2 | 26 | 48 |
| **Nulliparous > postpartum 9 weeks** |  |  |  |  |  |  |  |
| Superior medial gyrus, supplementary motor area, superior frontal gyrus | 8, 6, 32 | L/R | 1513 | 4.97 | -2 | 26 | 48 |
| **Nulliparous > postpartum 12 weeks** |  |  |  |  |  |  |  |
| Superior medial gyrus, supplementary motor area, superior frontal gyrus | 8, 6, 32 | L/R | 1043 | 4.7 | -2 | 26 | 48 |

Table S4. Brain regions showing gray matter volume increase in postpartum women throughout the postpartum period (t-contrast from random-effects GLM, p < .05, cluster-level FWE correction).

| **Anatomical Region** | **Brodmann** | **Side** | **Size** | **T** | **x** | **y** | **z** |
| --- | --- | --- | --- | --- | --- | --- | --- |
| **3 weeks > childbirth** |  |  |  |  |  |  |  |
| Middle occipital gyrus, middle temporal gyrus, precuneus gyrus, postcentral gyrus, precentral gyrus, middle frontal gyrus, inferior parietal gyrus, midcingulate cortex, superior parietal gyrus, supramarginal gyrus, superior frontal gyrus, cerebellum crus 1, fusiform gyrus, inferior frontal gyrus p. triangularis, inferior temporal gyrus, superior temporal gyrus, angular gyrus, superior occipital gyrus, cerebellum crus 2, inferior parietal lobule, cuneus, insula, supplementary motor area, rolandic operculum, cerebellum 6, anterior cingulate cortex, inferior frontal gyrus p. opercularis, cerebellum 8, rolandic operculum, inferior occipital gyrus, lingual gyrus, calcarine gyrus, superior medial frontal gyrus, paracentral lobule, medial orbital gyrus, cerebellum 7b, caudate nucleus, inferior frontal gyrus p. orbitalis, thalamus, Heschl gyrus, rectal gyrus, cerebellum 4 5, hippocampus, superior temporal pole, parahippocampal gyrus, middle orbital gyrus, amygdala, superior orbital gyrus, middle temporal pole, olfactory cortex, superior orbital gyrus, vermis 4 5 | 7, 6, 19, 40, 10, 9, 31, 13, 18, 39, 32, 22, 37, 3, 4, 8, 24, 21, 46, 5, 2, 41, 44, 20, 45, 11, 42, 43, 23, 47, 1, 30, 36, 17, 38, 25, 34, 28, 33, 29, 27, 35 | L/R | 200555 | 9.39 | -21 | -86 | 20 |
| **6 weeks >3 weeks** |  |  |  |  |  |  |  |
| Middle frontal gyrus, superior frontal gyrus, inferior frontal gyrus p. triangularis, precentral gyrus, inferior frontal gyrus p. orbitalis, middle orbital gyrus, inferior frontal gyrus o. opercularis, superior orbital gyrus, insula, superior medial gyrus, postcentral gyrus, rolandic operculum | 10, 9, 6, 8, 46, 11, 47, 45, 13, 44, 4, 32 | L/R | 15378 | 6.03 | -27 | 14 | 52 |
| Cerebellum crus 1, inferior parietal lobule, cerebellum crus 2, middle occipital gyrus, inferior occipital gyrus, postcentral gyrus, supramarginal gyrus, angular, middle temporal gyrus, superior temporal gyrus, fusiform gyrus, cerebellum 8, cerebellum 7b, cerebellum 6, rolandic operculum, lingual gyrus, superior parietal lobule | 40, 19, 39, 37, 2, 18, 42, 20, 13, 3, 21, 1, 4, 43, 22, 6, 41 | L | 13085 | 5.63 | -27 | -81 | -6 |
| Middle temporal gyrus, superior temporal gyrus, inferior temporal gyrus, angular gyrus, superior occipital gyrus, middle occipital gyrus, fusiform gyrus, superior parietal lobule, supramarginal gyrus, rolandic operculum, cerebellum crus 1, postcentral gyrus, inferior occipital gyrus, insula, precuneus, cerebellum 6 | 22, 21, 39, 7, 37, 41, 20, 13, 40, 19, 42, 43, 2, 1 | R | 6970 | 6.44 | 54 | -46 | -10 |
| Midcingulate cortex, superior medial gyrus, supplementary motor area, precuneus, paracentral lobule, anterior cingulate cortex, posterior cingulate cortex | 6, 31, 8, 32, 7, 24, 9, 5, 23 | L/R | 6420 | 5.74 | -3 | 6 | 56 |
| Superior frontal gyrus, middle frontal gyrus, superior orbital gyrus, middle orbital gyrus, superior medial gyrus, inferior frontal gyrus p. orbitalis, inferior frontal gyrus p. triangularis | 10, 11, 46, 47 | R | 2268 | 5.31 | 30 | 66 | 9 |
| Middle temporal gyrus, superior temporal gyrus | 21, 22 | L | 2054 | 6.13 | -64 | -26 | -3 |
| Middle occipital gyrus, superior parietal lobule, superior occipital gyrus, precuneus, cuneus, inferior parietal lobule | 19, 7, 39, 18, 31 | L | 1944 | 5.11 | -8 | -74 | 36 |
| Superior frontal gyrus, middle frontal gyrus, inferior frontal gyrus p. opercularis, precentral gyrus | 8, 9, 6 | R | 1311 | 4.88 | 21 | 16 | 48 |
| Caudate nucleus | - | L/R | 721 | 4.9 | -4 | 14 | 8 |
| **9 weeks > 6 weeks (p < .001)** |  |  |  |  |  |  |  |
| Inferior frontal gyrus p. triangular, inferior frontal gyrus p. opercularis, insula, rolandic operculum, superior temporal pole, middle frontal gyrus | 13, 34, 19, 12, 2 | L | 688 | 5.73 | -44 | 28 | 10 |
| **9 weeks > 6 weeks (p < .005)** |  |  |  |  |  |  |  |
| Inferior frontal gyrus p. triangular, inferior frontal gyrus p. opercularis, insula, rolandic operculum, precentral gyrus, postcentral gyrus, superior temporal gyrus, middle frontal gyrus, superior temporal pole | 13, 44, 22, 9, 46, 6, 45, 43, 4, 47 | L | 1822 | 5.73 | -44 | 28 | 10 |
| Superior medial gyrus, anterior cingulate cortex, supplementary motor area, midcingulate cortex, medial orbital gyrus | 32, 9, 10, 6, 24, 8 | L/R | 1593 | 4.01 | 0 | 16 | 45 |
| Parahippocampal gyrus, amygdala, superior temporal pole, insula, hippocampus, pallidum, olfactory cortex | 34, 28, 38, 13, 47, 25 | R | 1329 | 3.75 | 24 | 6 | -26 |
| **12 weeks > 6 weeks** |  |  |  |  |  |  |  |
| Superior medial frontal gyrus, superior frontal gyrus, supplementary motor area, middle frontal gyrus, anterior cingulate cortex, midcingulate cortex | 9, 8, 32, 6, 10 | L/R | 1809 | 4.64 | -2 | 18 | 47 |
| Inferior temporal gyrus, middle temporal pole, fusiform gyrus, amygdala, superior temporal pole, parahippocampal gyrus, middle temporal gyrus, hippocampus | 20, 21, 38, 28 | R | 808 | 4.74 | 63 | -9 | -36 |
| Inferior temporal gyrus, middle temporal gyrus, middle temporal pole, superior temporal gyrus, superior temporal pole | 21, 20, 38 | R | 672 | 4.63 | -62 | -20 | -23 |
| Inferior temporal gyrus, middle temporal gyrus, superior temporal pole, middle temporal pole | 20, 21, 38 | L | 555 | 4.6 | -53 | -15 | -30 |


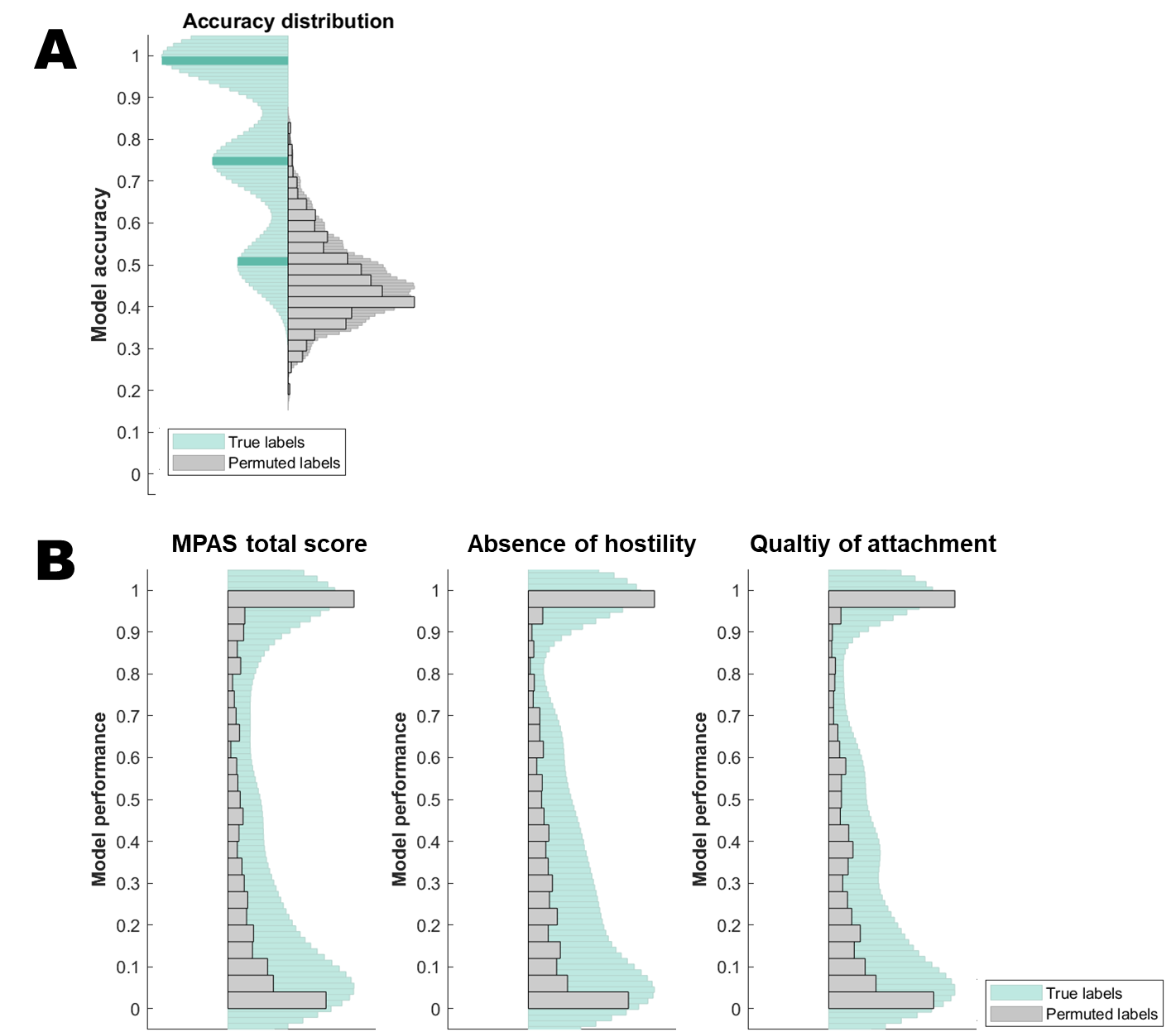


Figure S2. Density plots for the permutation test of the A) accuracy distribution and B) R2 distribution of the MPAS total score and the subscales absence of hostility and quality of attachment.

Table S5. Predictive regions with >1% contribution to the decision function for both the MPAS total score and the subscale absence of hostility

| **ROI Label** | **ROI weight (%)** | **ROI size (voxel)** | **Exp. Ranking** |
| --- | --- | --- | --- |
| **MPAS total score** |  |  |  |
| Vermis 8 | 2.01 | 528 | 116.55 |
| Cerebelum 7b L | 1.7717 | 863 | 114.4 |
| Cerebelum Crus1 L | 1.6553 | 5334 | 113.85 |
| Cerebelum 9 R | 1.6408 | 1320 | 110.55 |
| Cerebelum Crus2 L | 1.6007 | 4105 | 112.1 |
| Cerebelum 6 R | 1.5807 | 4096 | 111.55 |
| Cerebelum Crus1 R | 1.5434 | 4791 | 111.2 |
| Putamen L | 1.3488 | 2255 | 107.3 |
| Cerebelum Crus2 R | 1.3136 | 3901 | 105.7 |
| Cerebelum 7b R | 1.2629 | 692 | 104.25 |
| Precuneus L | 1.2556 | 7574 | 103.45 |
| Inferior occipital R | 1.2427 | 2411 | 101.4 |
| Cerebelum 8 L | 1.204 | 2619 | 101.75 |
| Paracentral lobule L | 1.1915 | 2490 | 101.4 |
| Thalamus R | 1.1836 | 2362 | 101.85 |
| Cerebelum 6 L | 1.1756 | 4087 | 100.6 |
| Superior temporal pole R | 1.1595 | 2085 | 100 |
| Vermis 6 | 1.1449 | 797 | 93.35 |
| Rectus R | 1.1086 | 1571 | 93.1 |
| Cerebelum 4 5 L | 1.0905 | 2715 | 94.25 |
| Superioral orbital frontal R | 1.0897 | 1352 | 92.55 |
| Olfactory L | 1.087 | 671 | 94.3 |
| Fusiform L | 1.0637 | 5282 | 90.7 |
| Middle temporal pole R | 1.0593 | 1810 | 90.7 |
| Amygdala L | 1.0593 | 487 | 89.1 |
| Putamen R | 1.059 | 2560 | 89.75 |
| Middle orbital frontal R | 1.0575 | 1583 | 88.6 |
| Superior temporal pole L | 1.0514 | 2708 | 90.45 |
| Supplementary motor area L | 1.0499 | 4720 | 88 |
| Cerebelum 3 L | 1.0343 | 314 | 84.3 |
| Middle temporal pole L | 1.0241 | 1524 | 88.9 |
| Cerebelum 10 R | 1.0213 | 286 | 82.9 |
| **Absence of hostility** |  |  |  |
| Amygdala R | 2.55 | 571 | 116.85 |
| Amygdala L | 1.77 | 487 | 113.85 |
| Middle temporal pole L | 1.66 | 1524 | 114.6 |
| Superior temporal pole R | 1.41 | 2085 | 111.45 |
| Cerebelum Crus2 L | 1.39 | 4105 | 109.8 |
| Cerebelum Crus1 R | 1.39 | 4791 | 110.8 |
| Cerebelum 9 R | 1.39 | 1320 | 108.4 |
| Thalamus L | 1.35 | 2420 | 109.2 |
| Vermis 7 | 1.34 | 458 | 106.75 |
| Inferior occipital R | 1.32 | 2411 | 105 |
| Supplementary motor area L | 1.29 | 4720 | 103.85 |
| Middle temporal pole R | 1.28 | 1810 | 106.1 |
| Paracentral Lobule L | 1.27 | 2490 | 102.75 |
| Cerebelum Crus2 R | 1.24 | 3901 | 100.25 |
| Cerebelum Crus1 L | 1.23 | 5334 | 101.25 |
| Cerebelum 6 L | 1.23 | 4087 | 102.15 |
| Cerebelum 3 L | 1.21 | 314 | 98.7 |
| Precuneus L | 1.17 | 7574 | 94.3 |
| Vermis 6 | 1.16 | 797 | 92.95 |
| Olfactory R | 1.16 | 643 | 95.05 |
| Cerebelum 7b R | 1.16 | 692 | 94.25 |
| Cerebelum 4 5 L | 1.16 | 2715 | 94.9 |
| Precentral L | 1.15 | 7671 | 94.2 |
| Angular L | 1.15 | 2739 | 93.25 |
| Insula R | 1.15 | 4160 | 94.15 |
| Cerebelum 6 R | 1.14 | 4096 | 95 |
| Cerebelum 7b L | 1.14 | 863 | 95.4 |
| Parahippocampal R | 1.1 | 2557 | 91.4 |
| Cerebelum 8 L | 1.09 | 2619 | 89 |
| Vermis 8 | 1.08 | 528 | 89.55 |
| Thalamus R | 1.07 | 2362 | 88.7 |
| Parahippocampal L | 1 | 2344 | 85.45 |
| Superior temporal pole L | 1 | 2708 | 85.4 |

Note. L = left hemisphere; R = right hemisphere.

Table S6. Cortical thickness differences of nulliparous women > postpartum women (t-contrast from random-effects GLM, p < .05, cluster-level FWE correction).

| **Anatomical region** | **Side** | **Size** | **Value** |
| --- | --- | --- | --- |
| **Nulliparous > Postpartum childbirth** |  |  |  |
| Rostral middle frontal gyrus, superior frontal gyrus, caudal middle frontal gyrus, inferior frontal gyrus pars opercularis, precentral gyrus, medial orbitofrontal gyrus, inferior frontal gyrus pars orbitalis, postcentral gyrus, frontal pole | L | 3371 | 6 |
| Supramarginal gyrus, middle temporal gyrus, inferior parietal gyrus, superior temporal gyrus | L | 1615 | 5.6 |
| Rostral middle frontal gyrus, superior frontal gyrus, medial orbitofrontal gyrus, caudal middle frontal gyrus, lateral orbitofrontal gyrus, frontal pole | R | 1180 | 6.1 |
| Superior parietal lobule, precuneus | L | 924 | 6.2 |
| Superior parietal lobule, inferior parietal lobule | R | 859 | 5.2 |
| Supramarginal gyrus, inferior parietal lobule | R | 761 | 5.3 |
| Superior temporal gyrus, middle temporal gyrus, transverse temporal gyrus | R | 751 | 4.7 |
| Superior frontal gyrus, caudal anterior cingulate gyrus | R | 682 | 4.7 |
| Precuneus, paracentral lobule, superior parietal lobule, superior frontal gyrus | R | 654 | 5.2 |
| Postcentral gyrus, inferior frontal gyrus pars opercularis, precentral gyrus, supramarginal gyrus, insula | R | 603 | 5.4 |
| Inferior parietal lobule, lateral occipital gyrus, superior parietal lobule | L | 423 | 5 |
| Superior frontal gyrus | L | 338 | 5 |
| Caudal middle frontal gyrus, rostral middle frontal gyrus, precentral gyrus | R | 316 | 5.2 |
| Superior temporal gyrus, transverse temporal gyrus | L | 262 | 4.6 |
| Inferior temporal gyrus, middle temporal gyrus, fusiform gyrus | R | 260 | 5.7 |
| Inferior frontal gyrus pars triangularis, rostral middle frontal gyrus | R | 167 | 5.4 |
| Fusiform gyrus | R | 157 | 4.9 |
| Lateral occipital gyrus, fusiform gyrus, lingual gyrus | L | 143 | 4.4 |
| Isthmus of cingulate gyrus, posterior cingulate cortex, precuneus | L | 135 | 4.6 |
| Superior parietal lobule | R | 132 | 4.7 |
| Lingual gyrus, isthmus of cingulate gyrus, precuneus | L | 119 | 5.7 |
| Supramarginal gyrus, postcentral gyrus | L | 110 | 4 |
| Superior temporal gyrus | R | 95 | 4.1 |
| **Nulliparous > Postpartum 3 weeks** |  |  |  |
| Superior parietal lobule, precuneus | L | 380 | 4.8 |
| Supramarginal gyrus, inferior parietal lobule | L | 372 | 5.1 |
| Rostral middle frontal gyrus, inferior frontal gyrus pars triangularis | L | 299 | 5.2 |
| Superior parietal lobule, inferior parietal lobule | R | 287 | 4.3 |
| Middle temporal gyrus | L | 286 | 4.6 |
| Precentral gyrus, inferior frontal gyrus pars opercularis | L | 284 | 4.3 |
| Superior frontal gyrus | L | 214 | 4.5 |
| Inferior temporal gyrus, middle temporal gyrus, fusiform gyrus | R | 212 | 5 |
| Superior frontal gyrus, medial orbitofrontal gyrus, frontal pole, rostral middle frontal gyrus | L | 200 | 4.1 |
| Inferior parietal gyrus, supramarginal gyrus | R | 194 | 4.4 |
| Medial orbitofrontal gyrus, rostral middle frontal gyrus, lateral orbitofrontal gyrus, frontal pole | R | 187 | 4.3 |
| Superior frontal gyrus, caudal middle frontal gyrus, rostral middle frontal gyrus | R | 186 | 4.8 |
| Middle temporal gyrus, superior temporal gyrus | R | 166 | 4 |
| Rostral middle frontal gyrus, superior frontal gyrus, caudal middle frontal gyrus | L | 156 | 4.5 |
| Rostral middle frontal gyrus | R | 142 | 4.4 |
| Supramarginal | R | 126 | 4.4 |
| Inferior frontal gyrus pars opercularis, precentral gyrus | R | 120 | 4.3 |
| Inferior parietal lobule | L | 119 | 4.2 |
| Caudal middle frontal gyrus, rostral middle frontal gyrus | L | 118 | 4.3 |
| Medial orbitofrontal gyrus | L | 107 | 4.7 |
| Postcentral gyrus | R | 98 | 4.1 |
| Rostral middle frontal gyrus | R | 94 | 4.7 |
| **Nulliparous > Postpartum 6 weeks** |  |  |  |
| Superior parietal lobule | L | 251 | 4.3 |
| Precentral gyrus, inferior frontal gyrus pars opercularis | L | 239 | 4 |
| Superior parietal lobule, inferior parietal lobule | R | 223 | 4.5 |
| Inferior parietal lobule, supramarginal gyrus | L | 142 | 4.4 |
| Middle temporal gyrus | L | 113 | 4.1 |
| Inferior parietal lobule, supramarginal gyrus | R | 109 | 4.1 |
| Superior frontal gyrus, caudal middle frontal gyrus, rostral middle frontal gyrus | R | 104 | 4.6 |
| Rostral middle frontal gyrus | L | 95 | 4.5 |
| Inferior frontal gyrus pars opercularis, precentral gyrus | R | 92 | 4 |
| Precuneus | R | 90 | 4.9 |
| **Nulliparous > Postpartum 9 weeks** |  |  |  |
| Superior parietal lobule, precuneus | L | 238 | 4.1 |
| Inferior parietal lobule, supramarginal gyrus | L | 191 | 4.6 |
| Precentral gyrus ,inferior frontal gyrus pars opercularis | L | 184 | 4.2 |
| Inferior parietal lobule, supramarginal gyrus | R | 105 | 4.2 |
| Inferior temporal gyrus | R | 103 | 4.5 |
| **Nulliparous > Postpartum 12 weeks** |  |  |  |
| Inferior frontal gyrus pars opercularis, precentral gyrus | R | 145 | 4 |
| Medial orbitofrontal gyrus, lateral orbitofrontal gyrus | L | 92 | 4.2 |

Note. Atlas labeling was performed according to the Desikan-Killiany atlas.

Table S7. Cortical thickness alteration in postpartum women throughout the postpartum period (t-contrast from random-effects GLM, p < .05, cluster-level FWE correction).

| **Anatomical region** | **Side** | **Size** | **T-value** |
| --- | --- | --- | --- |
| **3 weeks > childbirth** |  |  |  |
| Superior parietal lobule, precuneus, inferior parietal lobule, postcentral gyrus, supramarginal gyrus, lateral occipital gyrus, paracentral lobule cuneus, isthmus of cingulate gyrus | R | 3580 | 6.6 |
| Precuneus, superior parietal lobule, inferior parietal lobule, paracentral lobule, superior frontal gyrus, cuneus, isthmus of cingulate gyrus, caudal anterior cingulate gyrus, posterior cingulate gyrus | L | 2751 | 5.4 |
| Supramarginal gyrus, superior temporal gyrus, postcentral gyrus, insula, precentral gyrus, transverse temporal gyrus, inferior frontal gyrus pars opercularis | R | 1015 | 5.4 |
| Rostral middle frontal gyrus, superior frontal gyrus | L | 699 | 4.7 |
| Precentral gyrus, caudal middle frontal gyrus, inferior frontal gyrus p. opercularis, postcentral gyrus, rostral middle frontal gyrus | R | 597 | 6.2 |
| Supramarginal gyrus, postcentral gyrus, superior parietal gyrus | L | 518 | 6.7 |
| Inferior frontal gyrus pars triangularis, inferior frontal gyrus pars opercularis, precentral gyrus, postcentral gyrus, insula | L | 494 | 4.9 |
| Precentral gyrus, superior frontal gyrus, postcentral gyrus | R | 485 | 4.6 |
| Fusiform gyrus, inferior temporal gyrus, lateral occipital gyrus, lingual gyrus | L | 469 | 5 |
| Superior frontal gyrus | R | 405 | 4.3 |
| Supramarginal gyrus, superior temporal gyrus, postcentral gyrus | L | 331 | 4.5 |
| Superior frontal gyrus, rostral middle frontal gyrus | R | 262 | 5 |
| Caudal middle frontal gyrus, precentral gyrus | L | 246 | 4.4 |
| Posterior cingulate gyrus, superior frontal gyrus, paracentral lobule | R | 167 | 4.5 |
| Lateral occipital gyrus, fusiform gyrus | R | 154 | 4.9 |
| Inferior frontal gyrus pars triangularis, lateral orbital frontal gyrus, inferior frontal gyrus pars opercularis | R | 153 | 5.1 |
| Precentral gyrus, caudal middle frontal gyrus | L | 141 | 4.7 |
| Fusiform gyrus, lingual gyrus | R | 135 | 4.3 |
| Superior parietal lobule, postcentral gyrus | L | 135 | 5.1 |
| Rostral middle frontal gyrus | R | 127 | 4.8 |
| Lateral orbitofrontal gyrus, insula, inferior frontal gyrus pars orbitalis | L | 120 | 4.7 |
| Lateral orbitofrontal gyrus, inferior frontal gyrus pars orbitalis | L | 100 | 3.8 |
| Postcentral gyrus, supramarginal gyrus | R | 99 | 3.7 |
| Superior temporal gyrus, insula | L | 87 | 4.4 |
| inferior temporal gyrus, middle temporal gyrus, lateral occipital gyrus | R | 85 | 4.4 |
| **6 weeks > 3 weeks** |  |  |  |
| Superior parietal lobule | L | 92 | 4.4 |
| Superior frontal gyrus | L | 136 | 4 |
| Inferior frontal gyrus pars orbitalis, lateral orbital frontal gyrus, rostral middle frontal gyrus | R | 162 | 4.8 |
| Caudal middle frontal gyrus, rostral middle frontal gyrus | R | 165 | 4.6 |
| **9 weeks > 6 weeks** |  |  |  |
| Rostral middle frontal gyrus, inferior frontal gyrus pars opercularis | R | 138 | 4.3 |
| Middle temporal gyrus, inferior temporal gyrus | L | 85 | 4.3 |
| Superior frontal gyrus | R | 83 | 4.7 |
| **12 weeks > 9 weeks (p < .005)** | |  |  |
| Postcentral gyrus, precentral gyrus, supramarginal gyrus | R | 260 | 3.5 |
| **Childbirth > 12 weeks** |  |  |  |
| Lingual gyrus | L | 120 | 4.5 |
| Medial orbitofrontal gyrus, lateral orbitofrontal gyrus | R | 94 | 5.2 |

Note. Atlas labeling was performed according to the Desikan-Killiany atlas.

Table S8. Sulcus depth alteration in postpartum women throughout the postpartum period (t-contrast from random-effects GLM, p < .05, cluster-level FWE correction).

| Anatomical region | Side | Size | T-value |
| --- | --- | --- | --- |
| 3 weeks > childbirth |  |  |  |
| Supramarginal gyrus, postcentral gyrus, superior parietal lobule, precentral gyrus, inferior parietal lobule | R | 1794 | 4.9 |
| Caudal middle frontal gyrus, rostral middle frontal gyrus, precentral gyrus, inferior frontal gyrus pars opercularis, inferior frontal gyrus pars triangularis, superior frontal gyrus | R | 1191 | 4.8 |
| Inferior frontal gyrus pars triangularis, inferior frontal gyrus pars opercularis, insula, precentral gyrus, lateral orbitofrontal gyrus, postcentral gyrus | R | 582 | 4.7 |
| Supramarginal gyrus, postcentral gyrus, superior parietal lobule, inferior parietal lobule | L | 547 | 4.5 |
| Superior temporal gyrus, supramarginal gyrus, transverse temporal gyrus, insula | L | 522 | 5.3 |
| Inferior parietal lobule, middle temporal gyrus, superior temporal gyrus, supramarginal gyrus | L | 520 | 5.1 |
| Superior temporal gyrus, transverse temporal gyrus, insula | R | 229 | 5 |
| Caudal middle frontal gyrus, precentral gyrus, inferior frontal gyrus pars opercularis | L | 223 | 4.6 |
| Rostral middle frontal gyrus, inferior frontal gyrus pars triangularis | L | 204 | 5.6 |
| Lingual gyrus, isthmus cingulate, precuneus | L | 198 | 4.3 |
| Precentral gyrus, postcentral gyrus | L | 151 | 4.1 |
| Precentral gyrus, postcentral gyrus | L | 128 | 4.4 |
| Rostral middle frontal gyrus | L | 124 | 4.2 |
| Supramarginal gyrus, inferior parietal lobule, | L | 121 | 4.2 |
| Precentral gyrus, inferior frontal gyrus pars opercularis | L | 118 | 3.9 |
| Rostral middle frontal gyrus | L | 108 | 4.4 |
| Lingual gyrus, precuneus, pericalcarine gyrus | R | 102 | 3.9 |
| Superior parietal lobule, postcentral gyrus | L | 101 | 3.8 |
| Precuneus, superior parietal lobule | L | 91 | 3.9 |
| 6 weeks > 3 weeks |  |  |  |
| Inferior parietal lobule, middle temporal gyrus, supramarginal gyrus, superior temporal gyrus, superior parietal gyrus, inferior temporal gyrus | R | 2136 | 5.2 |
| Inferior frontal gyrus pars opercularis, inferior frontal gyrus pars triangularis, rostral middle frontal gyrus, precentral gyrus, inferior frontal gyrus pars orbitalis, caudal middle frontal gyrus, insula, lateral orbitofrontal gyrus | L | 1686 | 5.7 |
| Fusiform gyrus, lingual gyrus, inferior temporal gyrus, lateral occipital gyrus, parahippocampal gyrus | L | 1262 | 5.2 |
| Supramarginal gyrus, superior temporal gyrus, transverse temporal gyrus, insula, postcentral gyrus | R | 949 | 5 |
| Inferior parietal lobule, middle temporal gyrus, supramarginal gyrus, lateral occipital gyrus, | L | 638 | 4.8 |
| Rostral middle frontal gyrus, inferior frontal gyrus pars orbitalis, inferior frontal gyrus pars triangularis | R | 388 | 5.1 |
| Supramarginal gyrus, postcentral gyrus | L | 367 | 3.9 |
| Precentral gyrus, rostral middle frontal gyrus, caudal middle frontal gyrus, inferior frontal gyrus pars opercularis | R | 347 | 4.4 |
| Precentral gyrus, paracentral lobule, postcentral gyrus | L | 189 | 4.5 |
| Superior parietal gyrus, precuneus | R | 156 | 4.1 |
| Rostral middle frontal gyrus | R | 139 | 3.9 |
| 9 weeks > 6 weeks |  |  |  |
| Superior temporal gyrus, transverse temporal gyrus | L | 379 | 4.6 |
| Supramarginal gyrus, superior temporal gyrus | L | 142 | 3.8 |
| 12 weeks > 9 weeks (p < .005) |  |  |  |
| Inferior frontal gyrus pars opercularis, caudal middle frontal gyrus, rostral middle frontal gyrus, precentral gyrus, inferior frontal gyrus pars triangularis | R | 189 | 3.1 |


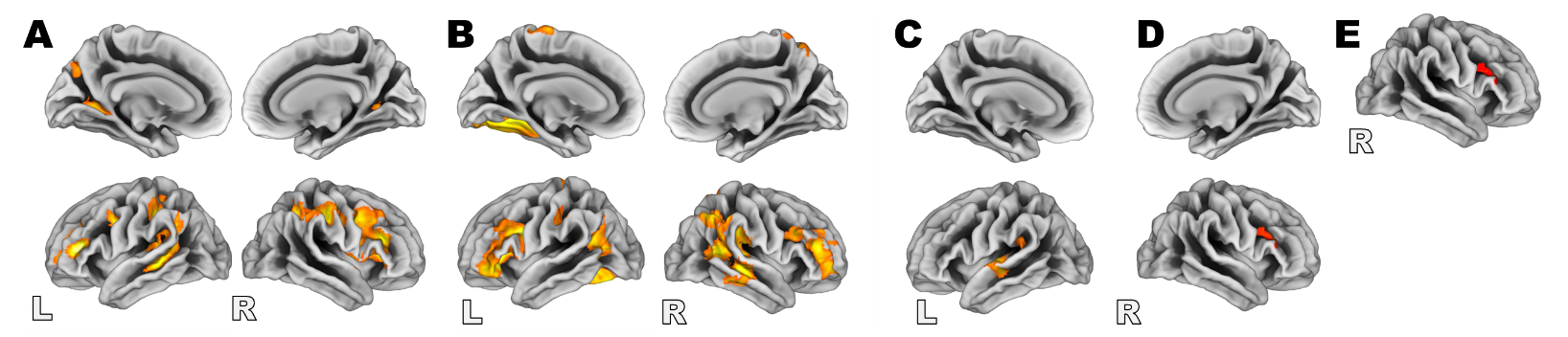


Figure S3. Sulcus depth decrease in postpartum from A) childbirth to 3 weeks postpartum, B) 3 weeks to 6 weeks postpartum, C) 6 weeks to 9 weeks postpartum, D) 9 weeks to 12 weeks postpartum.
